# Psychometric properties of the Turkish version of the Vestibular Migraine Patient Assessment Tool and Handicap Inventory

**DOI:** 10.55730/1300-0144.5876

**Published:** 2024-07-22

**Authors:** Deniz Uğur CENGİZ, Feyza İNCEOĞLU, Ercan KARABABA, Bülent SATAR, Hatice Seyra ERBEK, Tuba BAYINDIR, Büşra KURTCU, Sanem Can ÇOLAK, Mehmet Kadir ERCAN, Emre SÖYLEMEZ, Metin Yüksel AKYILDIZ, Yüce İSLAMOĞLU, Bengi ARSLAN, Sümeyye DEMİREL BİRİŞİK, Emre Akgün ÖZDEMİR, İsmail DEMİR, Ahmet ADIGÜZEL

**Affiliations:** 1 Department of Audiology, Faculty of Health Sciences, İnönü University, Malatya, Turkiye; 2 Department of Biostatistic, Faculty of Medicine, Malatya Turgut Özal University, Malatya, Turkiye; 3 Department of Audiology, Gülhane Faculty of Health Sciences, University of Health Sciences, Ankara, Turkiye; 4 Department of Otorhinolaryngology Head and Neck Surgery, Faculty of Medicine, University of Health Sciences, Ankara, Turkiye; 5 Department of Otorhinolaryngology Head and Neck Surgery, Faculty of Medicine, Lokman Hekim University, Ankara, Turkiye; 6 Department of Otorhinolaryngology Head and Neck Surgery, Faculty of Medicine, İnönü University, Malatya, Turkiye; 7 Departmant of Otorhinolaryngology Head and Neck Surgery, Institute of Health Sciences, İnönü University, Malatya, Turkiye; 8 Department of Audiology, Ankara Bilkent City Hospital, Ankara, Turkiye; 9 Department of Audiology, Vocational School of Health Services, Karabük University, Karabük, Turkiye; 10 Department of Otolaryngology Head and Neck Surgery, Darıca Farabi Teaching and Research Hospital, Kocaeli, Turkiye; 11 Department of Otolaryngology Head and Neck Surgery, Ankara City Hospital, Ankara, Turkiye; 12 Departmant of Audiology, Faculty of Health Sciences, Bingöl University, Bingöl, Turkiye; 13 Department of Audiology, İnönü University Turgut Özal Medical Center, Malatya, Turkiye; 14 Department of Neurology, Faculty of Medicine, İnönü University, Malatya, Turkiye

**Keywords:** Otology, audiology, vertigo, balance, neurology

## Abstract

**Background/aim:**

There are insufficient tools to assist in the diagnosis and treatment of vestibular migraine. Hence, the aim of this study was to perform the Turkish adaptation of the Vestibular Migraine Patient Assessment Tool and Handicap Inventory (VM-PATHI).

**Materials and methods:**

After the language and content validity was completed, a pilot study was conducted. Exploratory factor analysis (EFA) and confirmatory factor analysis (CFA) were conducted to test construct validity, and as a result of the validity analyses, Cronbach’s alpha internal consistency coefficient and test-retest analyses were conducted for reliability.

**Results:**

In the study, in which 289 participants were evaluated, the Kaiser–Meyer–Olkin coefficient was calculated as 0.903. The percentage of variance explained by the EFA was 67.246% and the range of factor load change was 0.433–0.828. The scale structure was tested with CFA and the model was confirmed with adequate goodness of fit index values. The Cronbach’s alpha internal consistency coefficient of the scale was 0.931.

**Conclusion:**

The VM-PATHI is a valid and reliable tool for the subjective evaluation of vestibular migraine in Türkiye.

## 1. Introduction

Vertigo is one of the most common complaints seen in otorhinolaryngology and neurology clinics [1]. Vestibular migraine (VM) is one of the most common causes of episodic vertigo and it is a type of migraine in which symptoms of dizziness and imbalance are combined with migraine symptoms of headache, vomiting, phonophobia, photophobia, and visual aura [2]. Currently, VM accounts for 4%–10% of diagnoses in clinics specializing in specific areas such as dizziness and headache, but this rate reflects the differences in inclusion criteria between various studies [3,4]. Migraine is considered to be the most common neurologic cause of vertigo. However, due to the inadequacies of objective and subjective diagnostic tools, clinicians have difficulty in the differential diagnosis of VM, which may cause the incidence of VM to be low [3]. The Barany Association published definite and probable VM diagnostic criteria [4]. However, there are no biomarkers that will help monitor the treatment process and evaluate the severity of the disease [2–5]. It was aimed to make an important contribution to the literature by establishing Turkish validity and reliability of the Vestibular Migraine Patient Assessment Tool and Handicap Inventory (VM-PATHI), which will help the diagnosis of VM. Considering that VM is a commonly encountered condition in audiology, otorhinolaryngology, neurology, and psychiatry clinics, the importance of the Turkish validity and reliability of the scale increases even greater. The scale, which was developed for the subjective evaluation of VM, is unique in the literature and will be adapted to a different language for the first time herein.

## 2. Materials and methods

### 2.1. Study design and sampling

Scale validity and reliability studies are designed methodologically. The sample size was calculated as at least 10 times the number of items (there are 25) in the scale. Accordingly, 289 sampling units were reached in the planned period for the research [6]. The voluntary sampling method was used in the selection of the sampling units.

The study included individuals aged 18–60 years who had no vestibular system-related diseases other than VM, met the diagnostic criteria for VM as defined by the Barany Association, and were native Turkish speakers. Individuals under 18 and over 60 years of age with active vestibular system diseases other than VM were excluded from the study. The research was conducted with face-to-face interviews between November 2022 and July 2023. The study had a multicenter design and data were collected from five centers and six clinics.

### 2.2. Data collection tools

A sociodemographic data form and the VM-PATHI were used in the study. A Sociodemographic Data Form, consisting of 8 items (age, sex, institution, educational status, marital status, time of onset of vestibular symptoms, time of onset of auditory symptoms, and Barany Society VM diagnostic criteria) was administered to the participants.

The VM-PATHI, developed by Sharon et al. [5], consists of 25 items and 6 subdimensions. The scale’s internal consistency Cronbach’s alpha coefficient was determined as 0.92. In the scoring of the scale, a 5-point Likert-type scoring is used, in which 0 = none, 1 = mild, 2 = moderate, 3 = severe, and 4 = as severe as possible. The scale yields a total score between 0 and 100. In the study, two separate groups of 50 patients and 18 controls were recruited and the study was completed with 68 participants. The subdimensions of the scale are: cognition (items 4, 13, 14, 17, 20, and 25), emotion/sense of being overwhelmed (items 9, 15, 16, 18, and 24), disequilibrium/central audiovestibular disturbance (DCAD) (items 1, 2, 7, 11, 12 and), anxiety (items 5, 6, and 23), motion sensitivity (items 3, 10, and 21), and headache equivalents (items 8, 19, and 22) [5]. To conduct the Turkish validity and reliability study of the scale, permission was obtained from the corresponding author, Jeffrey D. Sharon via e-mail.

### 2.3. Multivariate normal distribution analysis

Since exploratory factor analysis (EFA) and confirmatory factor analysis (CFA) are multivariate analysis methods, multivariate normal distribution was checked in the database [7]. Three hundred and five data forms were collected, outliers and missing observations were checked, and the study dataset was prepared. Of these, 16 questionnaire forms were excluded from the study due to these reasons and based on the Mahalanobis distance criterion; therefore, the study was completed with 289 questionnaire forms [8]. The observation farthest from the centroid (Mahalanobis Distance) value was calculated using AMOS as 7.104. The assumption was met, as the calculated multivariate normal distribution coefficient was less than 8 [9].

### 2.4. Validity analyses

Validity analyses of the scale adaptations were completed in three stages. First, the language validity analysis was conducted for the question form. In the second stage, the questions were sent for expert opinion for content validity. In the last stage, EFA and CFA were applied for the construct validity analysis.

#### 2.4.1. Scale language validity analysis

The study began by obtaining language adaptation permission for the VM-PATHI. The VM-PATHI was sent to two different language experts and translated into Turkish. While one of the translators completed the translation with the blinded method, the other completed the translation while being informed about the purpose of the study. Both questionnaires were compared, and the most descriptive items were included in the question pool. The questionnaire was sent to a third language expert, a native English speaker with a good command of Turkish. The third expert was asked to translate the questionnaire into English and the new scale was compared with the original scale, and the scale was finalized by preserving the integrity of meaning. After the language validity phase was completed, the scale was sent to different experts for their expert opinions [10].

#### 2.4.2. Scope validity and pilot study

After the language validity was completed, the questionnaire was sent to 14 experts in the field and they were asked to score the questions on the form between 1 and 3 (1 = the item should be removed, 2 = the item should be corrected, and 3 = the item is sufficient). The Kendall W index was calculated by evaluating the score forms received from the experts. According to the responses of the 14 experts, the index value was 0.089 (p = 0.192). As a result of the expert opinions, the VM-PATHI question pool was statistically sufficient and had a measurement level [11].

For the pilot study, 30 participants were recruited. In the first evaluation of the VM-PATHI, it was observed that the questions were understandable, and the participants were able to respond quickly and easily.

#### 2.4.3. Psychometric testing of the VM-PATHI and construct validity

For the psychometric analyses, first, the scale structure was determined by applying EFA within the scope of construct validity. The scale model prepared with EFA was rechecked and verified with CFA and the final model of the VM-PATHI was obtained.

### 2.5. Ethics committee permission

Approval was obtained from the University’s Institute of Health Sciences Non-Interventional Clinical Research Ethics Committee (Decision number: 2022/3927) and all the individuals participating in the study.

### 2.6. Data analysis

IBM SPSS Statistics for Windows 26.0 (IBM Corp., Armonk, NY, USA) was used for the Cronbach’s alpha internal consistency coefficient and test-retest analyses and EFA. AMOS 24 was preferred for the CFA. p < 0.05 was accepted as statistically significant. The descriptive statistics were expressed as the mean standard ± deviation (SD), numbers, and percentages.

## 3. Results

The demographic information of the participants is given in Table 1. According to the diagnostic criteria of the Barany Association, 40.8% of the participants were diagnosed as definite VM and 59.2% were probable VM. Of the participants, 63.7% were female and 36.3% were male. The mean age was 43.34 ± 11.5 years. The mean duration of onset of vestibular symptoms was 37.39 ± 39.43 days and the mean duration of onset of auditory symptoms was 27.7 ± 32.58 days. Vestibular symptoms started earlier than auditory symptoms (Table 1).

### 3.1. EFA

As a result of the EFA analysis, Kaiser–Meyer–Olkin (KMO), and Bartlett’s Test of Sphericity (BTS) values were calculated. The KMO coefficient was 0.903 and the BTS value was 4066.743. The fact that the calculated values were statistically within the desired range showed that the sample size and structure of the study were suitable for EFA [10].

The factor loadings and % variance explained values calculated as a result of the EFA are given in Table 2. The total explained variance ratio for the VM-PATHI, which consists of 25 items and 6 subdimensions, was 67.246% (Table 2).

The first subdimension of the scale consists of items 4, 13, 14, 17, 20, and 25 expressing the concept of cognition, and the explained variance ratio was 19.228. The factor loading values of the subdimension varied between 0.433 and 0.828. The second subdimension of the VM-PATHI consists of items 9, 15, 16, 18, and 24 expressing the concept of emotion/sense of being overwhelmed, and the explained variance ratio was 15.117. The factor loading values of the subdimension varied between 0.488 and 0.836. The third subdimension of the VM-PATHI consists of items 1, 2, 7, 11, and 12 expressing the concept of DCAD, and the explained variance ratio was 10.724. The factor loading values of the subdimension varied between 0.423 and 0.739. The fourth subdimension of the VM-PATHI consists of items 5, 6, and 23 expressing the concept of anxiety, and the explained variance ratio was 8.598. The factor loading values of the subdimension varied between 0.538 and 0.782. The fifth subdimension of the VM-PATHI consists of items 3, 10, and 21 expressing the concept of motion sensitivity, and the explained variance ratio was 8.266. The factor loading values of the subdimension varied between 0.486 and 0.618. The sixth subdimension of the VM-PATHI consists of items 8, 19, and 22 expressing the concept of headache equivalents, and the explained variance ratio was 5.314. The factor loading values of the subdimension varied between 0.447 and 0.657 (Table 2).

### 3.2. CFA

**Figure 1:**
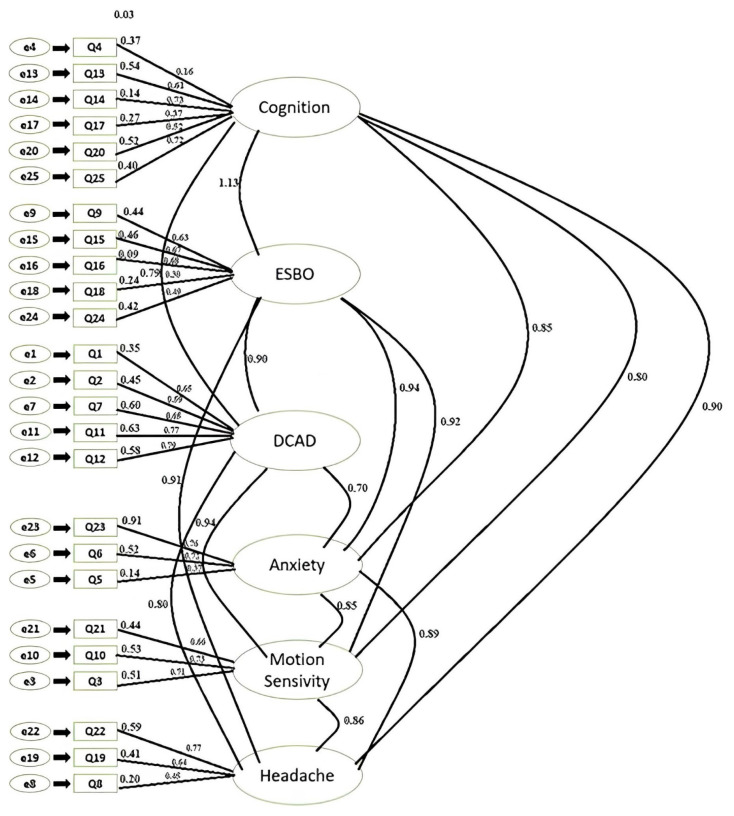
VM-PATHI first stage CFA model. (ESBO: emotion/sense of being overwhelmed, DCAD: disequilibrium/central audiovestibular disturbance).

CFA was applied to validate the scale model, consisting of 6 subdimensions and 25 questions after the EFA [12]. The first scale model is shown in Figure 1.

**Figure 2:**
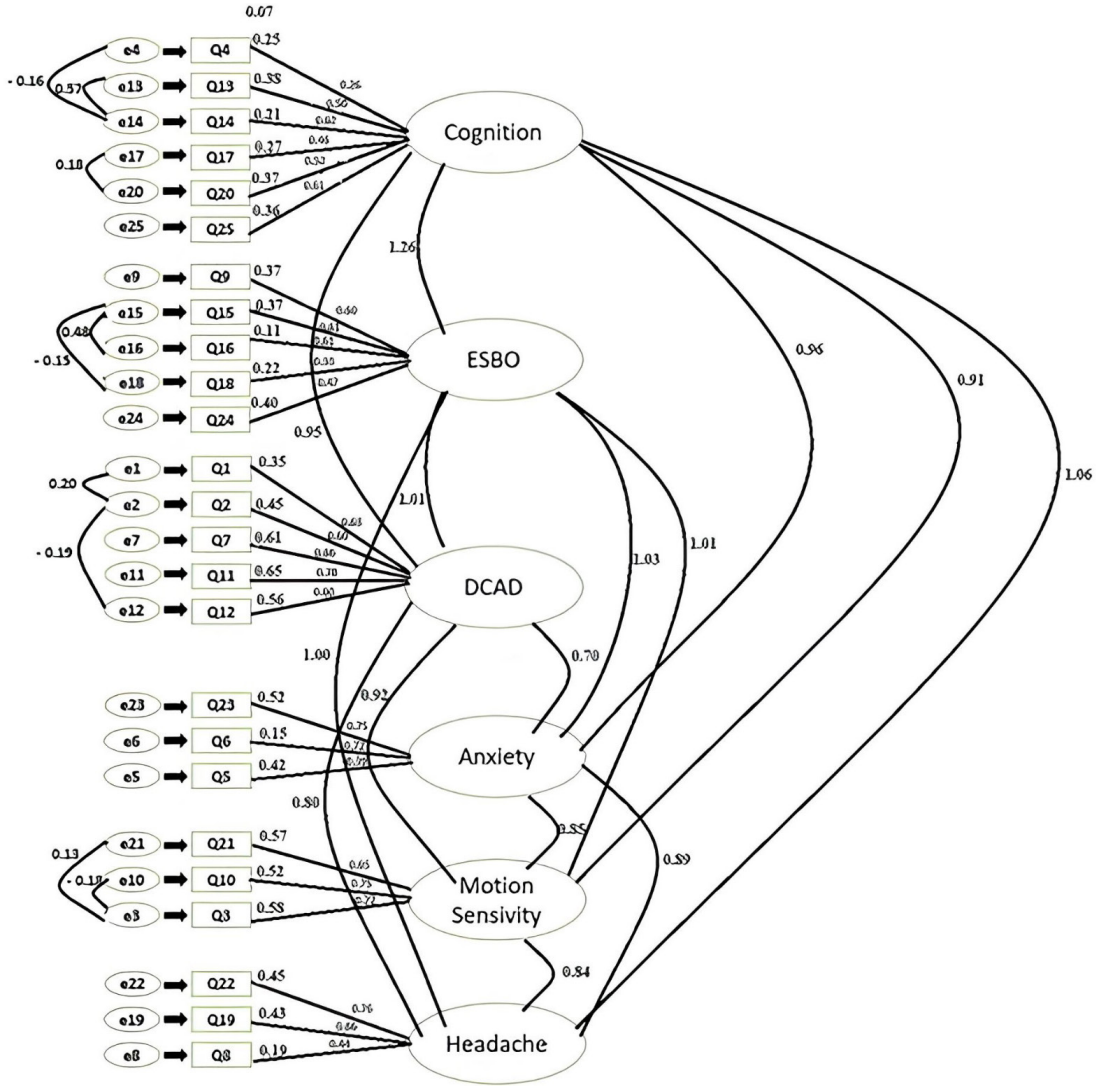
VM-PATHI modified CFA model.

The calculated goodness of fit index values of the model were χ2 chi-squared goodness of fit (CMIN): 1513.125 and χ2/df 5.820, normed fit index (NFI): 0.600, incremental fit index (IFI): 0.682, comparative fit index (CFI): 0.679, goodness of fit index (GFI): 0.557, and root mean square error of approximation (RMSEA): 0.129 (Figure 1). Model modification was applied because the calculated values were not at the desired level, and there were measurement errors and latent constructs [13]. In the control of the modification coefficients, covariances between the residual terms belonging to the same subdimension were plotted and a new model was obtained. The pairs of error terms with modification were e4–e14, e13–e14, e17–e20, e15–e16, e15–e18, e3–e10, and e3–e21. A diagram of the new scale model is given in Figure 2.

The goodness of fit index values calculated for the new VM-PATHI model were χ2: 1083.567, χ2/df 4.317, CFI: 0.901, NFI: 0.905, GFI: 0.917, IFI: 0.903, and RMSEA: 0.671. The decrease in the χ2/df and RMSEA values and increase in the CFI, NFI, GFI, and IFI values, which were higher than 0.90, showed that the established VM-PATHI model was statistically sufficient (Figure 2). The validity analyses for the VM-PATHI were completed and it was found that the model, consisting of 6 subdimensions and 25 questions, was statistically valid.

### 3.3. Reliability

For the reliability analyses, Cronbach’s alpha internal consistency coefficient and test-retest analyses were applied. The item total correlation and Cronbach’s alpha coefficients calculated for the VM-PATHI and its subdimensions are given in Table 3.

Cronbach’s alpha values were calculated as 0.812 for cognition, 0.784 for emotion/sense of being overwhelmed, 0.737 for disequilibrium/central audio vestibular disturbance, 0.718 for anxiety, 0.708 for motion sensitivity, and 0.701 for headache equivalents. The total Cronbach’s alpha coefficient was 0.931 (Table 3). Scale item correlation coefficient values ranged between 0.293 and 0.689, and since there were no items with a value lower than 0.20, no questions were eliminated [14].

### 3.4. Test-retest reliability

To calculate the test-retest reliability of the scale, the VM-PATHI was administered to 60 people at 15-day intervals and comparisons of both results are given in Table 4.

Cognition, emotion/sense of being overwhelmed, DCAD, anxiety, motion sensitivity, and headache equivalents subscales and the scale total did not show statistically significant differences in both measurements and were invariant over time. A highly statistically significant relationship was found between the answers received at the two different times (Table 4).

### 3.5. Scale score calculation

Receiver operating characteristic (ROC) analysis was used to calculate the prediction scores for the scale and its subdimensions. The values of the 289 participants included in the study and the cut-off values calculated for each subdimension and the total score of the scale are given in Table 5.

Cut-off values were calculated as 11 for the cognition subscale, 9 for the emotion sense of being overwhelmed subscale, 7 for the disequilibrium/central audio vestibular disturbance and anxiety subscales, and 6 for the motion sensitivity and headache equivalents subscales. The cut-off value for the total score of the scale was 48. Any participant with a score of 48 and above had an increased likelihood of VM. As the score increased, the likelihood of having symptoms also increased (Table 5).

## 4. Discussion

There is a need in clinics for VM-specific subjective measurements that confirm the diagnosis of VM or show the severity of the disease. Marcus et al. [15] created a 7-item questionnaire formed as a structured interview to address this need. Cohen’s kappa reliability test was applied to test the reliability of this questionnaire, and the result was 0.75. Celebisoy et al. [16] developed an 8-item scale named the Vestibular Migraine Diagnosis Questionnaire. Their questionnaire was 83.3% compatible with clinical diagnosis, with a k coefficient of 0.666, retest k values between 0.71 and 0.87, sensitivity of 82.8%, and specificity of 83.9%. The VM-PATHI, which provides a more comprehensive assessment compared to these measurement tools in the literature, has high reliability is a disease-specific outcome measure created by Sharon et al. [5] to assess the severity of the disease in individuals diagnosed with VM. The VM-PATHI focuses on all symptoms of the disease, not just one such as dizziness. VM affects visits to neurology and otorhinolaryngology clinics due to the cooccurrence of symptoms of dizziness and ligament pain. In addition to these symptoms, vomiting, phonophobia, photophobia, visual aura, depression, and anxiety affect the quality of life of individuals [5]. Evaluating all the symptoms that may be seen in VM patients within the VM-PATHI subscales helps to distinguish VM from other diseases. The evaluation of symptoms and emotions such as stress, anxiety, sadness, avoidance of social situations, and the thought that life will not be normal again is important to determine the level of psychological impact of VM on the individual.

In the development of the VM-PATHI, which was adapted to Turkish herein by conducting validity and reliability analyses with data from multiple centers, the diversity of symptoms seen in individuals diagnosed with VM was taken into account. Sharon et al. [5] completed their study with 68 people, including 50 patients and 18 controls. The scale covers not only neurology and otorhinolaryngology but also audiology and psychiatry, as it includes all the symptoms of the disease together. In the present study, 289 data forms were included in the analysis, following the rule that the sample size should be at least 10 times the number of items in the scale. The Kendall W value calculated for content validity was statistically sufficient and data were obtained from the main sample, as there were no problems in the pilot study. As a result of the EFA applied to establish construct validity, KMO and BTS values were obtained. The KMO coefficient is used for the adequacy of the number of data and the minimum value it can take is 0.60. A high BTS value indicates that the prepared data set is suitable for EFA. Since the KMO value calculated for the scale (0.903) was 0.90, this meant that the sample size was very good. It enabled the evaluation of VM by offering a field of use. Sharon et al. [5] calculated the KMO value as 0.74.

The factor loadings of the scale varied between 0.433 and 0.828 and the total explained variance percentage was 72.078. The coefficient found was high and had an explanatory value for a scale consisting of 6 dimensions [10]. The factor structure was analyzed by considering that the factor loadings of the items in the scale should be at least 0.30. No items were eliminated due to low factor loadings [17].

Sharon et al. [5] did not use CFA in their analysis. However, CFA, which is one of the most important steps of establishing construct validity, was used in the current study and the scale structure, consisting of 6 subdimensions and a total of 25 items, was confirmed. The value of the χ2/df was 4.317, indicating model significance, while that for the RMSEA was 0.671, indicating the control of the number of samples, the GFI was 0.917 indicating the percentage of variance explained, and the CFI, NFI, and IFI values calculated for the general fit of the model were 0.901, 0.905, and 0.903, respectively. Good model fit was achieved with a decrease in the χ2/df (≤5) and an increase in the RMSEA (≤0.08), and GFI, CFI, NFI, and IFI (>0.90) values [18,19]. CFA is one of the subanalyses of structural equation modeling (SEM) analysis. In SEM analyses, more than one index is given and interpreted for model fit [20,21]. Sharon et al. [5] found Cronbach’s alpha coefficients of 0.87 for cognition, 0.82 for emotion/sense of being overwhelmed, 0.84 for DCAD, 0.70 for anxiety, 0.70 for motion sensitivity, 0.69 for headache equivalents, and 0.92 for the VM-PATHI in total. In the current study, the Cronbach’s alpha coefficients were 0.812 for cognition, 0.784 for emotion/sense of being overwhelmed, 0.737 for DCAD, 0.718 for anxiety, 0.708 for motion sensitivity, and 0.701 for headache equivalents. The total Cronbach’s alpha coefficient was 0.931. Scale item correlation coefficient values varied between 0.293 and 0.689, and since there were no items with a value lower than 0.20, no questions were eliminated [14]. Sharon et al. [5] recruited 25 participants for a test-retest study and calculated the retest Cronbach’s alpha coefficient as 0.90 and the item-total correlation coefficient change was between 0.366 and 0.410.

Cronbach’s alpha internal consistency coefficient was used for the reliability analysis of the scale. The Cronbach’s alpha coefficient varies between 0 and 1, and below 0.50 is considered unacceptable. When the coefficient value approaches 1, it indicates that the reliability level increases [22]. Increasing the number of items in the scale will increase the reliability coefficient [23]. If the number of items in the scale is small, a Cronbach’s alpha coefficient of 0.50 is assumed as acceptable [24]. In addition to reliability, test-retest analysis was applied to determine the invariance of the scale over time and 60 participants were included in the study [25].

The VM-PATHI consists of 6 subdimensions: cognition, emotion/sense of being overwhelmed, DCAD, anxiety, motion sensitivity, and headache equivalents.

The first of the scale subdimensions, cognition, includes decreased productivity at work, difficulty concentrating, difficulty remembering things, fatigue, fear of falling, and photophobia. In this group, vestibular disorders have been shown to cause cognitive impairment [26]. However, the reason for the inclusion of photophobia in this subdimension may be that the visual system at the cortical level responds more strongly to intense, repetitive, or prolonged stimulation as a result of central neuronal overstimulation, which is thought to be involved in migraine pathogenesis [27]. The second subdimension, emotion/depression, includes fear that life will never be normal again, dizziness in the presence of intense visual stimuli, sadness, social avoidance, and false sense of movement. This dimension is based on the depression caused by the disease. The third subdimension, imbalance/central audiovestibular disturbance, includes imbalance, difficulty climbing stairs, dizziness on movement, difficulty in walking, and phonophobia. The reason for the inclusion of phonophobia in this subdimension may be considered as the failure to elaborate minimal acoustic input as a result of the effect of migraine at the subcortical level [28]. The fourth subdimension, anxiety, includes stress, anxiety, and dizziness. Dizziness in this subscale may be related to VM-induced anxiety tendencies. The fifth subscale, motion sensitivity, includes motion sickness, dizziness, and nausea. Spinning in this group is not called true vertigo. This is because the dizziness seen in motion sickness is usually a physiological response to real or virtual motion stimuli that occur in individuals with a normal vestibular system [29]. Therefore, dizziness here is seen as a condition that develops similar to the pathophysiology of motion sickness. Finally, the sixth dimension, equivalent headache, includes ear pressure, head pressure, and headache. In VM, head pressure and ear pressure sensations are considered as headache equivalents.

In the study by Sharon et al. [5], no cut-off value was determined for the scale. However, in the current study, a ROC analysis cutoff value was determined for both the subdimensions and the VM-PATHI. The cutoff value for the VM-PATHI was 48 and the area under the ROC curve (AUC) value was 0.863. ROC analysis was performed to determine the estimation point for the parameters. The ROC curve gives the appropriate estimation point for the measurement tool and the sensitivity and specificity ratios are obtained in the decisions made based on this point. An AUC value of 0.5 indicates no discrimination; values between 0.5 and 0.7 indicate that the test discrimination power is statistically insignificant; values between 0.7 and 0.8 are acceptable; values between 0.8 and 0.9 are very good; and values above 0.9 are excellent. AUCs are within the desired range for parameter lengths [30]. The higher the score on the scale, the higher the predisposition to VM.

In the Turkish adaptation stages, language, content, and construct validity analyses, and reliability analyses were completed and a valid and reliable VM-PATHI scale in Turkish was obtained. This study, which was designed to help researchers in adaptations to different languages, has shown that the results can be generalized in terms of receiving data from different centers. The VM-PATHI is a measurement tool that can be used by clinicians as part of clinical decision support system for VM patients and can be used as the first step in the diagnosis of patients’ predisposition to VM.

## Figures and Tables

**Table 1:** Demographic information of participants.

Variable	Groups	Frequency	Percent
**Centers**	Center 1	58	20.1
	Center 2	100	34.6
	Center 3	55	19.0
	Center 4	44	15.2
	Center 5	32	11.1
**Sex**	Female	184	63.7
	Male	105	36.3
**Marital status**	Single	79	27.3
	Married	210	72.7
**Education level**	Illiterate	2	0.7
	Primary school	32	11.1
	Secondary school	25	8.7
	High school	88	30.4
	University	142	49.1
**VM diagnostic criteria**	Definite VM	118	40.8
	Probable VM	171	59.2
Total		289	100.0
	**Mean ± SD**	**Min–max**	
**Age**	43.34 ± 11.5	18–69	
**Time of onset of vestibular symptoms**	37.39 ± 39.43	1–240	
**Time of onset of auditory symptoms**	27.7 ± 32.58	1–240	

**Table 2:** Factor loadings and variance explained by the VM-PATHI.

Items	Cognition	ESBO	DCAD	Anxiety	MS	HE
**Q4**	0.828					
**Q13**	0.722					
**Q14**	0.787					
**Q17**	0.478					
**Q20**	0.433					
**Q25**	0.766					
**Q9**		0.488				
**Q15**		0.836				
**Q16**		0.796				
**Q18**		0.646				
**Q24**		0.658				
**Q1**			0.739			
**Q2**			0.524			
**Q7**			0.423			
**Q11**			0.547			
**Q12**			0.631			
**Q5**				0.782		
**Q6**				0.579		
**Q23**				0.538		
**Q3**					0.618	
**Q10**					0.556	
**Q21**					0.486	
**Q8**						0.447
**Q19**						0.657
**Q22**						0.562
**% variance explained**	**19.228**	**15.117**	**10.724**	**8.598**	**8.266**	**5.314**
***Total***	**67.246**					

Q: question, MS: motion sensitivity, HE: headache equivalents.

**Table 3:** Item total correlation and Cronbach’s alpha coefficient of the VM-PATHI.

Items	Cognition	ESBO	DCAD	Anxiety	MS	HE
**Q4**	0.293					
**Q13**	0.480					
**Q14**	0.604					
**Q17**	0.441					
**Q20**	0.559					
**Q25**	0.608					
**Q9**		0.673				
**Q15**		0.555				
**Q16**		0.589				
**Q18**		0.369				
**Q24**		0.555				
**Q1**			0.592			
**Q2**			0.577			
**Q7**			0.631			
**Q11**			0.687			
**Q12**			0.689			
**Q5**				0.636		
**Q6**				0.663		
**Q23**				0.635		
**Q3**					0.683	
**Q10**					0.603	
**Q21**					0.472	
**Q8**						0.387
**Q19**						0.607
**Q22**						0.606
**Cronbach’s alpha**	**0.812**	**0.784**	**0.737**	**0.718**	**0.708**	**0.701**
***Total***	**0.931**					

**Table 4:** Test-retest reliability of VM-PATHI.

Groups		Mean ± SD	Min–max	t value	p^a^ value	r value	p^b^ value
**Cognition**	Test	10.79 ± 3.54	4–17	−0.870	0.391	0.961	0.009*
	Retest	11.76 ± 4.78	3–22				
**ESBO**	Test	8.55 ± 3.18	2–16	0.269	0.790	0.941	0.001*
	Retest	8.28 ± 4.3	1–17				
**DCAD**	Test	7.38 ± 4.1	2–16	−0.497	0.623	0.865	0.001*
	Retest	8 ± 4.69	3–12				
**Anxiety**	Test	6.76 ± 2.31	1–11	1.344	0.190	0.715	0.001*
	Retest	5.97 ± 2.38	1–10				
**MS**	Test	5.79 ± 2.14	1–11	1.094	0.283	0.669	0.001*
	Retest	5.21 ± 2.38	1–10				
**HE**	Test	5.93 ± 2.15	1–11	−0.063	0.951	0.917	0.001*
	Retest	5.97 ± 2.04	19–77				
**VM-PATHI**	Test	45.21 ± 14.53	16–81	0.008	0.993	0.918	0.002*
	Retest	45.17 ± 17.53	0–0				

t: paired t test value, r: Pearson’s correlation coefficient, p^a^ > 0.05; there is no difference between pretest and posttest. p^b^ < 0.05; There is a very strong significant relationship between the two values.

**Table 5:** Descriptive statistics and cut off values for the VM-PATHI.

Scale	Mean ± SD	Min- Max (n=289)	Min-Max Scores to Receive from the Scale	Cut off	Sensitivity	Specificity	AUC
**Cognition**	11.23 ± 4.34	2–22	0–24	11	0.914	0.714	0.907
**ESBO**	8.58 ± 3.99	1–19	0–20	9	0.901	0.807	0.897
**DCAVB**	7.8 ± 4.18	0–19	0–20	7	0.875	0.716	0.849
**Anxiety**	6.71 ± 2.59	1–12	0–15	7	0.872	0.807	0.814
**MS**	5.65 ± 2.63	0–12	0–15	6	0.893	0.796	0.787
**HE**	6 ± 2.37	1–11	0–15	6	0.714	0.701	0.710
**VM-PATHI**	45.98 ± 17.37	9–90	0–100	48	0.809	0.719	0.863
